# Possible Role of Peroxynitrite in the Responses Induced by Fusicoccin in Plant Cultured Cells

**DOI:** 10.3390/plants10010182

**Published:** 2021-01-19

**Authors:** Massimo Malerba, Raffaella Cerana

**Affiliations:** 1 Dipartimento di Biotecnologie e Bioscienze, Università degli Studi di Milano-Bicocca, 20126 Milan, Italy; massimo.malerba@unimib.it; 2 Dipartimento di Scienze dell’Ambiente e della Terra, Università degli Studi di Milano-Bicocca, Piazza della Scienza 1, 20126 Milan, Italy

**Keywords:** *Acer pseudoplatanus* L. cultured cells, apoptosis, cell death, defense response, fusicoccin, reactive oxygen species, reactive nitrogen species, urate

## Abstract

Fusicoccin (FC) is a well-known phytotoxin able to induce in *Acer pseudoplatanus* L. (sycamore) cultured cells, a set of responses similar to those induced by stress conditions. In this work, the possible involvement of peroxynitrite (ONOO^−^) in FC-induced stress responses was studied measuring both in the presence and in the absence of 2,6,8-trihydroxypurine (urate), a specific ONOO^−^ scavenger: (1) cell death; (2) specific DNA fragmentation; (3) lipid peroxidation; (4) production of RNS and ROS; (5) activity of caspase-3-like proteases; and (6) release of cytochrome *c* from mitochondria, variations in the levels of molecular chaperones Hsp90 in the mitochondria and Hsp70 BiP in the endoplasmic reticulum (ER), and of regulatory 14-3-3 proteins in the cytosol. The obtained results indicate a role for ONOO^−^ in the FC-induced responses. In particular, ONOO^−^ seems involved in a PCD form showing apoptotic features such as specific DNA fragmentation, caspase-3-like protease activity, and cytochrome *c* release from mitochondria.

## 1. Introduction

Fusicoccin (FC) is a well-known phytotoxin produced by *Phomopsis amygdali* (previously named *Fusicoccum amygdali*), a host-specific pathogenic fungus of almond and peach trees. The trees infected by this pathogen show irreversible opening of the stomata with the consequent lack of gas exchanges regulation. Excessive transpiration from the leaf surface causes appearance of necrotic areas and wilting of leaves with consequent death of the plant [1]. Unlike other phytotoxin, the molecular mechanism underlying the effect of FC is well known. In fact, in all tested plant materials, FC induces a 14-3-3 protein-mediated activation of the plasma membrane H^+^-ATPase, the main electrogenic transporter of plant cell. The increased proton extrusion induced by FC causes hyperpolarization of the plasma membrane and alteration of ionic gradients, which are the basis of the effects of the phytotoxin that include stimulation of important physiological processes of plants such as cell distension and, interruption of seed dormancy and germination [1,2]. The identification of the FC receptor as a complex formed by 14-3-3 protein and plasma membrane H^+^-ATPase and the variety of effects reported for the phytotoxin prompted the scientists to investigate the possible existence of other FC receptors. Initially, FC binding sites were reported only in higher plants [3], but later binding sites for [^3^H]FC with affinity comparable with plant receptor were found first in *Xenopus leavis* embryos [4] and searched after in other organisms. In fact, 14-3-3s are regulative proteins present with numerous isoforms in all eukaryotic organisms [5]. These proteins are able to bind phosphorylated target proteins, thus modulating their activity, turnover, and sub-cellular localization. In this way, 14-3-3 proteins are involved in the regulation of several fundamental physiological processes such as cell cycle progression, gene transcription, apoptosis, and cancer [6]. Chemically, FC is a glycosylated diterpene. Since it has been well demonstrated that different terpenoids possess anti-cancer activity [7,8], several studies have been performed to test the ability of FC to affect tumor cells viability. Although the molecular mechanism underlying the effect is not known, it has been clearly demonstrated that FC can induce apoptosis in various tumor cell lines, breast cancer and glioblastoma cells included [9,10]. In addition, it has been shown that FC via 14-3-3 proteins induces in vivo regeneration of cortical embryonic neurons in model animals and in vitro stimulates the growth of damaged axon [11], suggesting a potential use of the phytotoxin against central nervous system diseases [12]. All these results strongly encourage new research to clarify the amazing properties of FC [13].

In sycamore (*Acer pseudoplatanus* L.) cultured cells, a material well characterized both biochemically and physiologically, in addition to inducing H^+^-ATPase activation, FC induces a set of stress responses. These responses include: (i) cell death, which in a fraction of dead cells shows apoptotic features (specific DNA fragmentation and cytochrome *c* release from mitochondria); (ii) stimulation of alternative respiration; (iii) production of hydrogen peroxide (H_2_O_2_) and nitric oxide (NO); and (iv) accumulation of regulative 14-3-3 proteins in the cytosol and of the heat shock protein (Hsp) 70 endoplasmic reticulum (ER)-resident molecular chaperone Binding Protein (BiP) with concomitant modification in the ER architecture ([14] and references therein). A regulative role of reactive oxygen species (ROS) and reactive nitrogen species (RNS) in many aspects of plant metabolism, responses to FC included, is well documented [15,16,17]. In particular, several papers show that NO is crucial for the adaptation response of plants to stress ([18] and references therein]). In fact, application of NO donors was able to protect plants against different stresses, while treatment with NO scavengers reverses this protective effect [19]. Interestingly, better protection against stress can be achieved by the interaction between ROS and RNS. For example, plants treated with low doses of NO and H_2_O_2_ are more resistant to stress than plants treated with the single reactive species, even at high concentration, and the reaction between NO and O_2_^−^ is a key component of NO removal strategy. This highlights the complexity of the connection between ROS and RNS in plants [18]. These observations prompted researchers to hypothesize that in plant cells too ROS and NO may react to form another reactive species already reported in animal cells, peroxynitrite (ONOO^−^). Unlike animals, where it is clearly implicated in the regulation of important physiological processes, apoptosis and cancer progression included [20], in plants, very little is known about a possible physiological role for ONOO^−^. In plants, low levels of ONOO^−^ are formed continuously by the metabolism of peroxisomes and photosynthesizing chloroplasts. Higher levels of ONOO^−^ can be synthesized in response to stress, a condition where the production of NO and ROS increases ([21] and references therein). Due to its capacity to hard react with lipids, nucleic acids, and proteins, ONOO^−^ may induce both oxidative and nitrosative modifications in various biological systems [22]. Plant cells can prevent cytotoxic accumulation of ONOO^−^ by an array of decomposition pathways that include ascorbic acid, metalloporphyrins, thiols, flavonoids, and peroxynitrite reductase [22]. This accounts for the potential utilization of ONOO^−^ as regulative molecule in plants, too ([23] and references therein).

In this work, the role of ONOO^−^ in some of the stress responses induced by FC in sycamore-cultured cells was investigated. To this goal, in both the presence and absence of 2,6,8-trihydroxypurine (urate), a specific scavenger of ONOO^−^ [24], we measured some of the FC-induced responses. In particular, we measured cell death, specific DNA fragmentation, lipid peroxidation, production of RNS and ROS, activity of caspase-3-like proteases, release of cytochrome *c* from mitochondria, variations in the levels of molecular chaperones Hsp90 in the mitochondria and Hsp70 BiP in the ER, and of regulatory 14-3-3 proteins in the cytosol.

## 2. Results

### 2.1. Effect of FC and Urate on RNS Accumulation in the Cells

**Figure 1:**
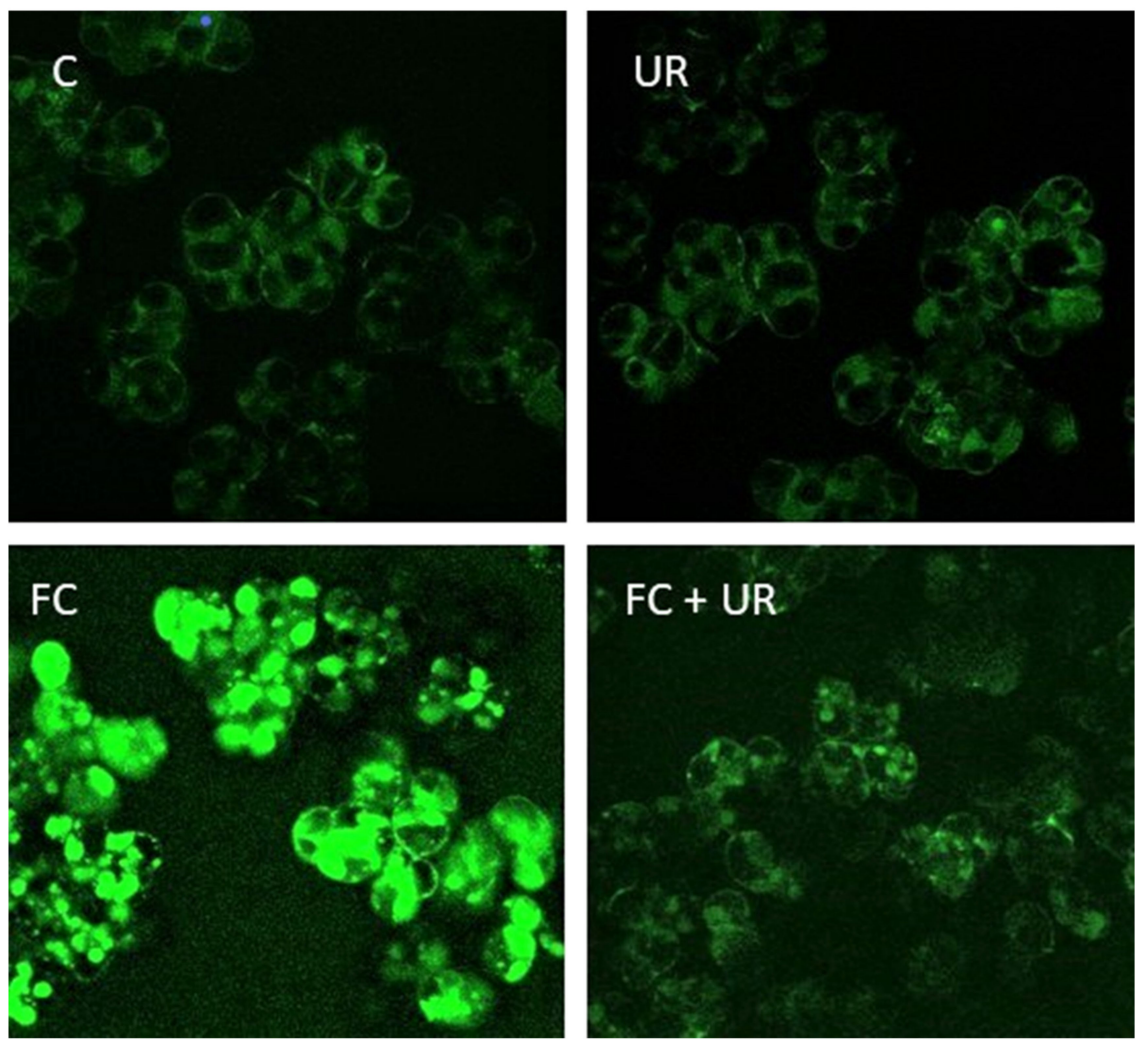
Effect of FC and urate on RNS accumulation in the cells. C, control cells; UR, cells treated with 1 mM urate; FC, cells treated with 20 µM FC; FC + UR, cells treated with 20 µM FC in the presence of 1 mM urate. Time of treatment 6 h.

With the aim of evaluating correctly the role of ONOO^−^ in the FC-induced responses, we preliminary tested the effect of urate on RNS accumulation in the cells. Figure 1 shows that the cells treated with FC show the bright green fluorescence due to RNS accumulation. Interestingly, this fluorescence is almost totally absent in the cells treated with FC in the presence of urate. These observations and the reported specificity of urate [24] make this chemical appropriate to investigate the role of ONOO^−^ in the FC-induced responses of sycamore cells.

### 2.2. Effect of FC and Urate on Cell Viability and on Accumulation of TUNEL-Positive Cells

**Figure 2:**
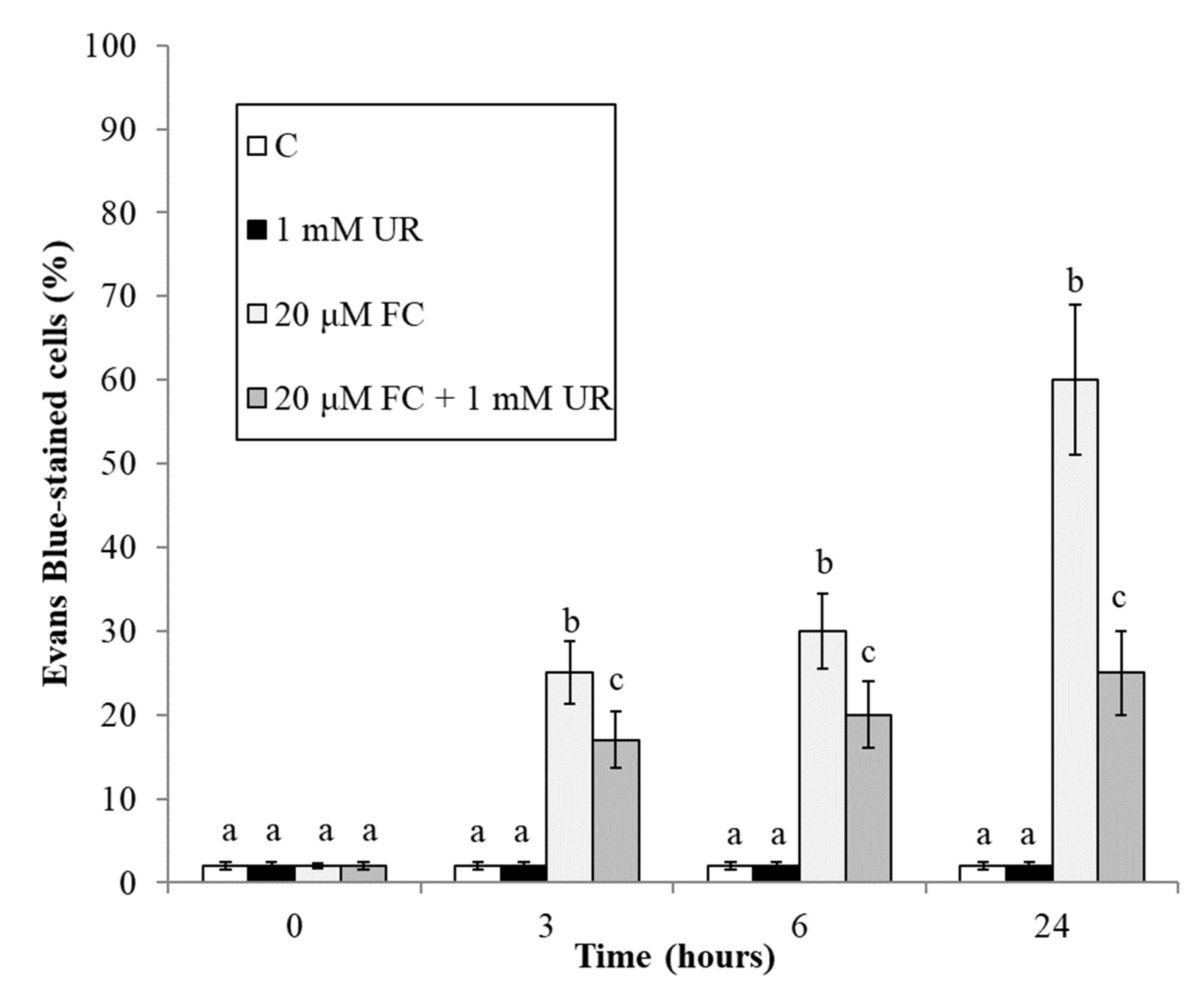
Effect of FC and urate on accumulation of dead cells in the cultures. Means ± SD (n ≥ 9) are shown. Different letters show significant differences among treatments at each time (Tukey HSD test, *p* ≤ 0.05).

Figure 2 reconfirms [25] that FC induces a progressive accumulation of dead cells in the cultures. At all experimental times, urate is able to reduce this accumulation.

**Figure 3:**
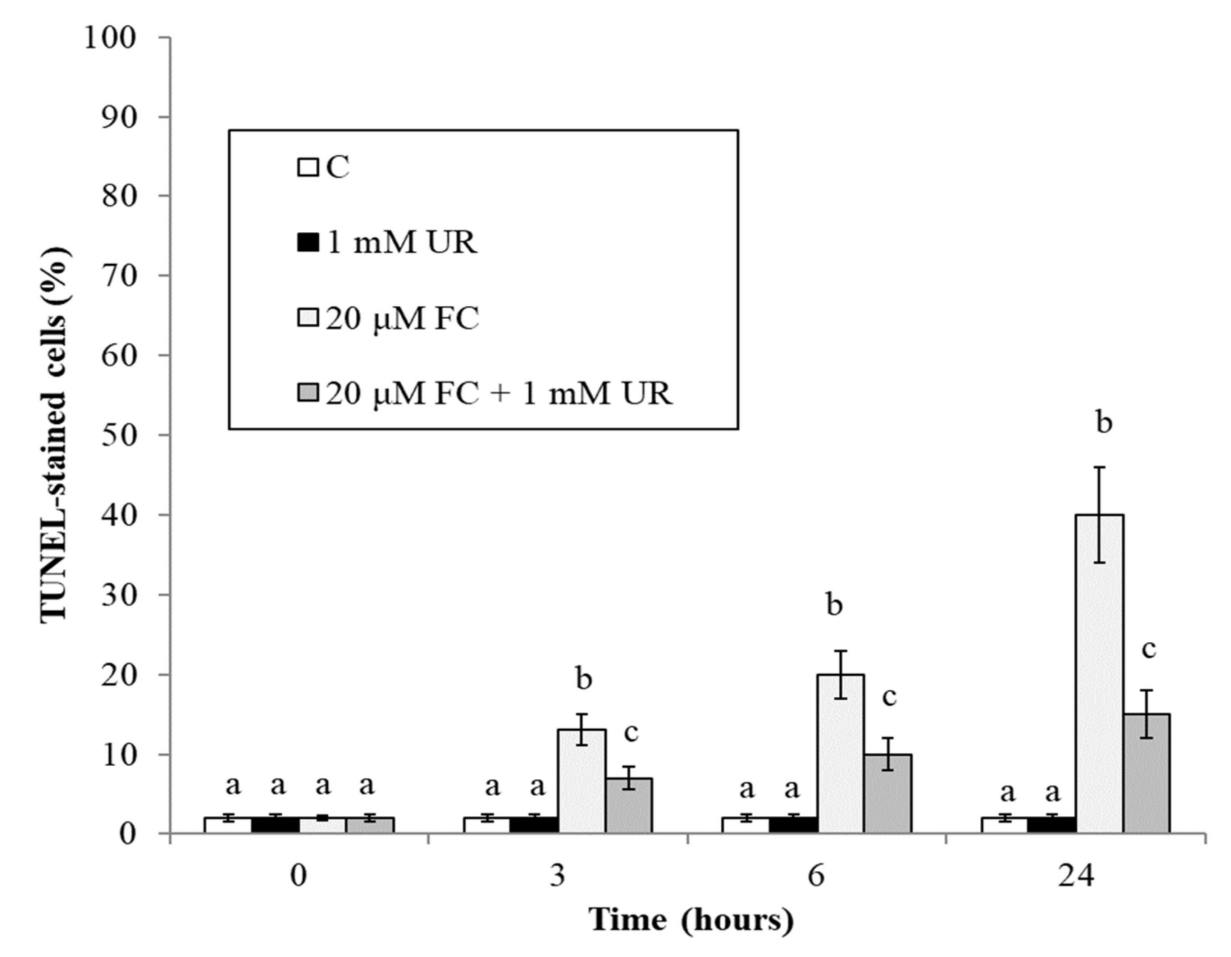
Effect of FC and urate on the appearance of TUNEL-positive cells in the cultures. Means ± SD (n ≥ 9) are shown. Different letters show significant differences among treatments at each time (Tukey HSD test, *p* ≤ 0.05).

Figure 3 reconfirms [25] that FC also induces the appearance of cells positive to the TUNEL reaction, a procedure that by measuring specific DNA cleavage is considered a good method to evidence the percentage of cells undergoing programmed cell death with apoptotic features [26]. At all experimental times, urate reduces the percentage of cells positive to the TUNEL reaction.

### 2.3. Effect of FC and Urate on Accumulation of O_2_^−^ and Malondialdehyde and on Caspase-3-Like Activity

**Figure 4:**
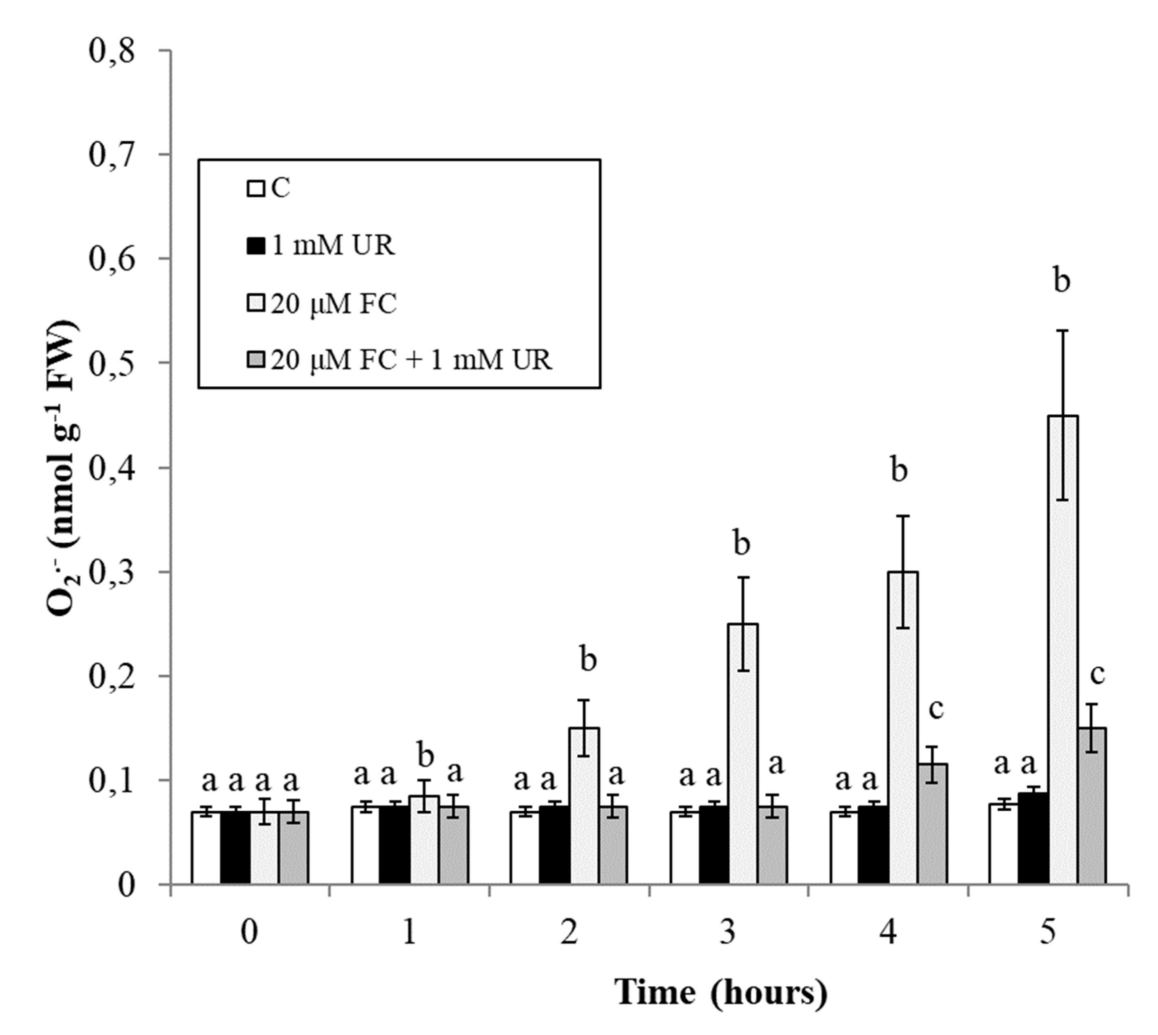
Effect of FC and urate on O_2_^−^ accumulation in the culture medium. Means ± SD (n ≥ 9) are shown. Different letters show significant differences among treatments at each time (Tukey HSD test, *p* ≤ 0.05).

O_2_*^−^*^.^ is a highly reactive ROS, responsible for the induction of important oxidative stress [18]. Treatment with FC (Figure 4) causes a progressive accumulation of O_2_*^−^*. This accumulation is almost totally prevented by urate, at least at the first experimental times (up to 4 h).

**Figure 5:**
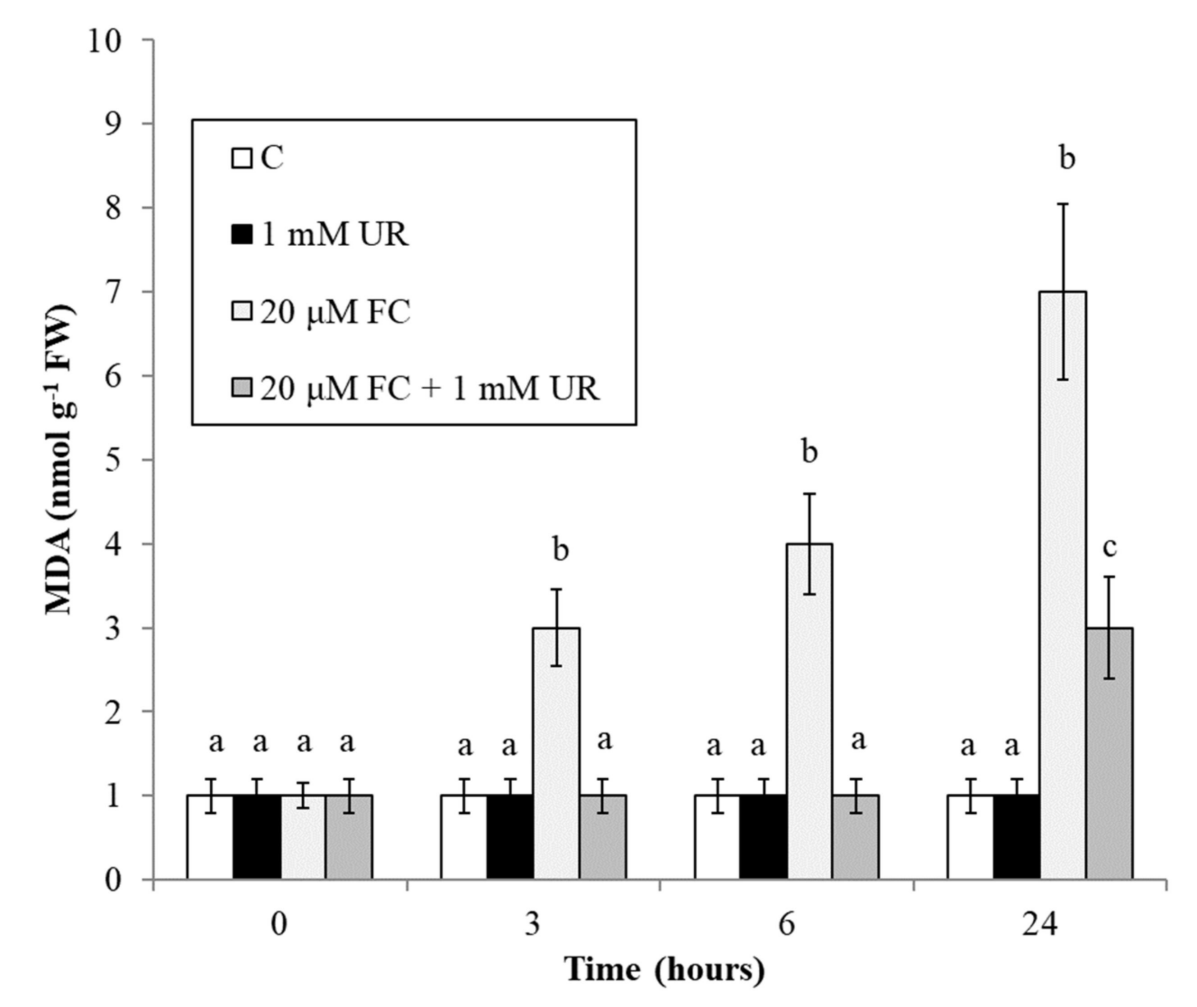
Effect of FC and urate on MDA accumulation. Means ± SD (n ≥ 9) are shown. Different letters show significant differences among treatments at each time (Tukey HSD test, *p* ≤ 0.05).

At cell level, peroxidation of membrane lipids is one of the main effects of oxidative stress, and its degree can be evaluated by determining the level of malondialdehyde (MDA), a byproduct of polyunsaturated fatty acids oxidation. Figure 5 shows that the level of MDA of the control cells is low, constant, and not influenced by urate. At each time, FC causes a considerable production of MDA that is totally prevented by urate (up to 6 h, at least).

**Figure 6:**
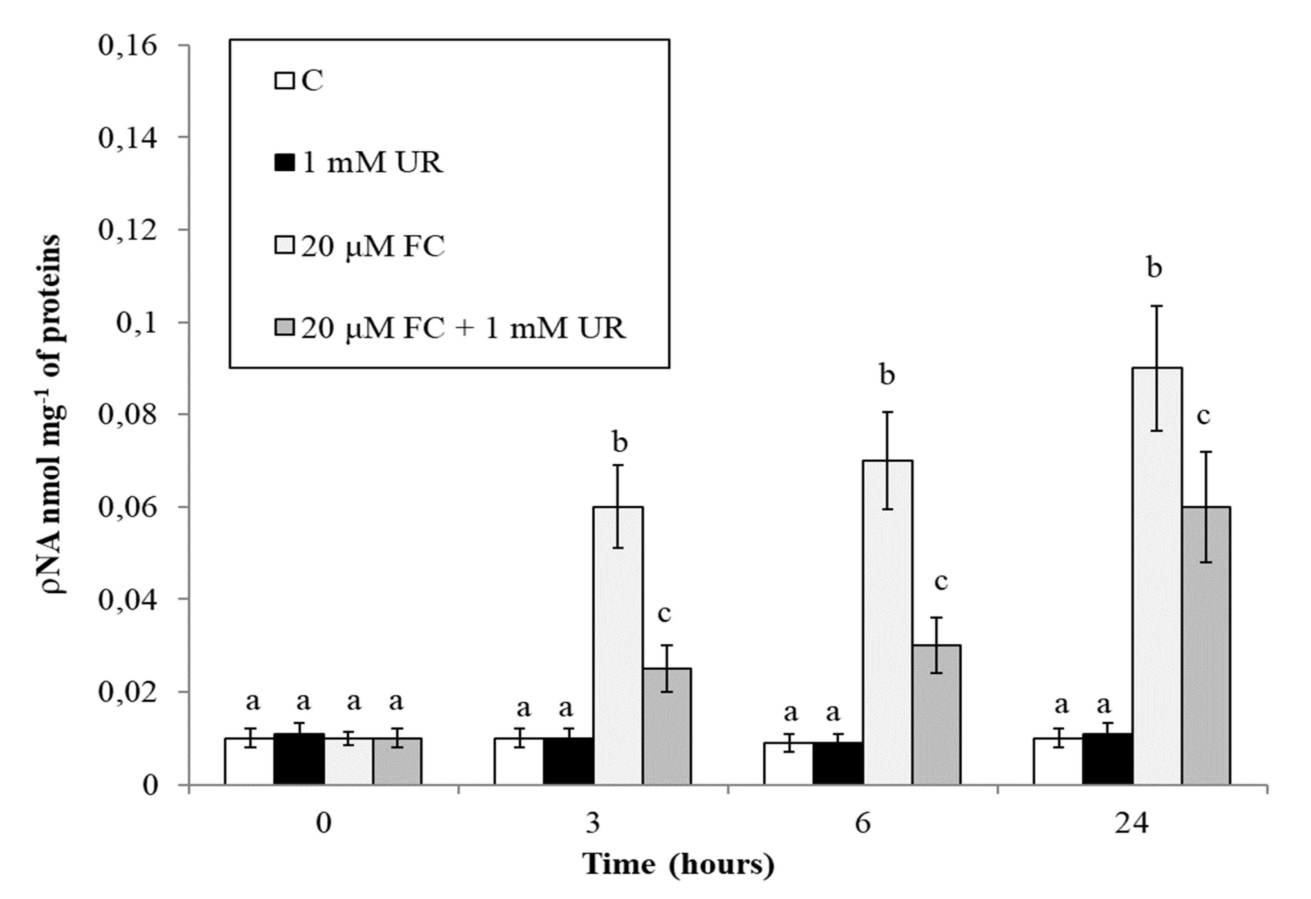
Effect of FC and urate on caspase-3-like activity. Means ± SD (n ≥ 6) are shown. Different letters show significant differences among treatments at each time (Tukey HSD test, *p* ≤ 0.05).

To characterize further the effect of urate on FC-induced cell death, we analyzed caspase-3-like proteases activity, another typical marker of PCD with apoptotic features that is often present during plant PCD [26]. Figure 6 shows that urate strongly reduces the FC-induced marked increase in this activity, at least at the first experimental times (up to 6 h).

### 2.4. Effects of FC and Urate on Stress-Related Proteins

Finally, by gel blotting experiments, we analyzed the effect of FC and urate on the levels of some stress-related proteins. In particular, we measured the levels of mitochondrial Hsp90, molecular chaperones that control the activity of different substrates, of Binding Protein (BiP), an Hsp70 present in the endoplasmic reticulum that accumulates under different stress conditions, and of regulative 14-3-3s, proteins involved in many processes of plant and animal cells, including cell death. In addition, we measured the release of cytochrome *c* from the mitochondrion, a marker of apoptotic death in animals and plants ([27] and references therein).

**Figure 7:**
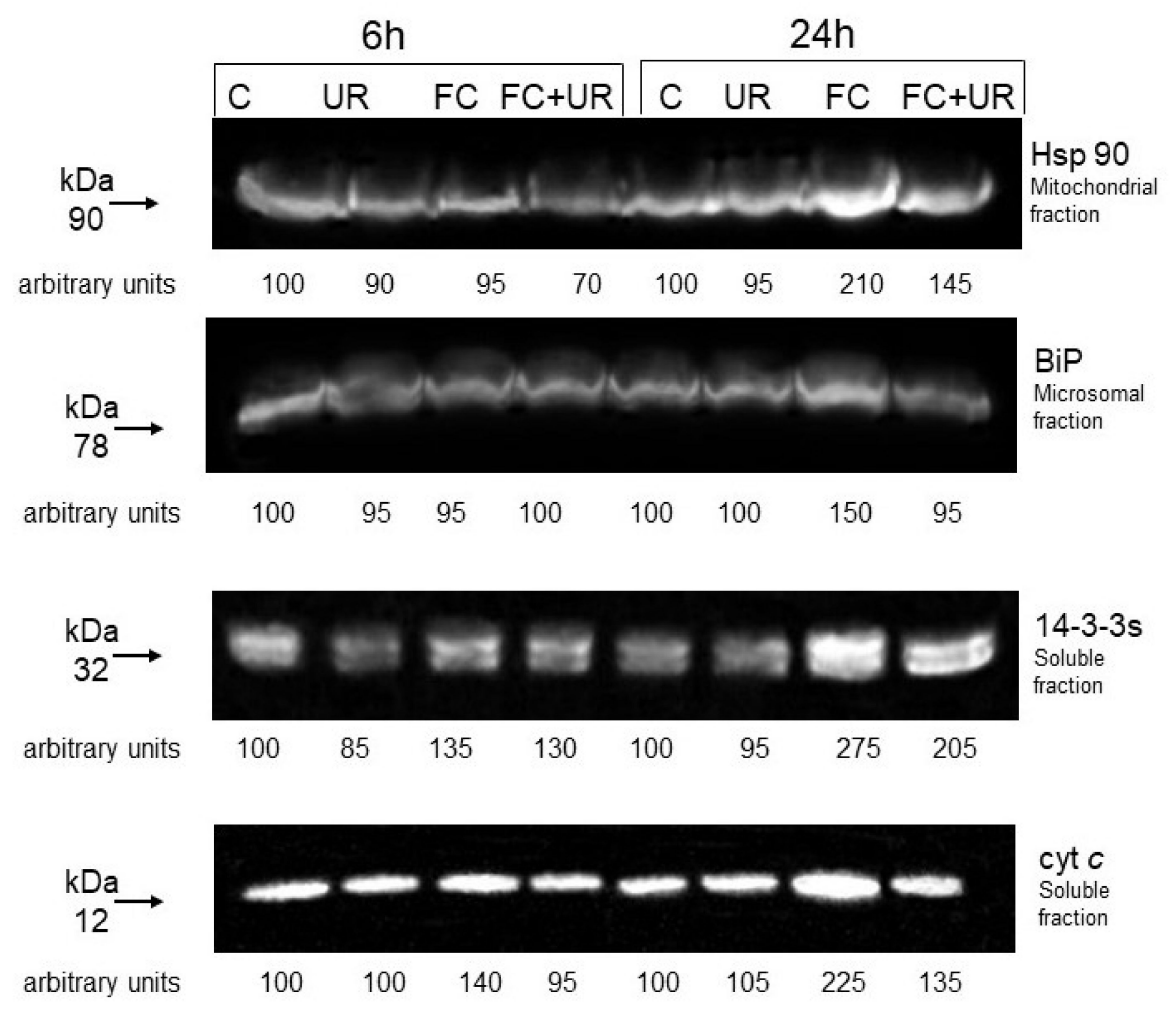
Effect of FC and urate on the levels of stress-related proteins. C, control; UR, cells + 1 mM urate; FC, cells treated with 20 µM FC; FC + UR, cells treated with 20 µM FC + 1 mM UR. Image and densitometric results of a typical experiment are presented. Fifty milligrams of proteins were run in each lane. At each experimental time, an arbitrary value of 100 was assigned to the quantity of immunodecorated protein of the controls. The experiment was repeated three times in duplicate. The variability did not exceed 20%.

Figure 7 confirms the previously reported effects of FC on BiP, 14-3-3s and cytochrome *c* levels [28] and shows that FC also affects the level of Hsp90. Urate diminishes the accumulations of Hsp90, BiP, and 14-3-3s elicited by the phytotoxin. Interestingly, at both experimental times, the release of cytochrome *c* from mitochondria induced by FC is almost totally prevented by urate.

## 3. Discussion

With the aim to clarify the possible role of ONOO^−^ as signaling molecule in plant cells, in this work, we tested the effect of urate, a specific ONOO^−^ scavenger, on some of the responses induced by FC in cultured cells. In particular, in both the absence and presence of the scavenger, we measured the effect of FC on cell death, positivity to the TUNEL reaction, production of RNS and ROS, lipid peroxidation, activity of caspase-3-like proteases, release of cytochrome *c* from mitochondria, variations in the levels of molecular chaperones Hsp90 in the mitochondria and Hsp70 BiP in the ER, and of 14-3-3 proteins in the cytosol. The urate concentration utilized in this work was chosen based on previous investigations conducted in cultured plant cells. In this experimental system 1 mM urate affected the responses induced by heat stress without decreasing cell vitality [27] and totally suppressed ONOO^−^ generation induced by INF1, the major elicitin secreted by the late blight pathogen *Phytophthora infestans*, with only slight effect on tyrosine nitration of proteins [29].

### 3.1. Effect of FC and Urate on ONOO^−^ Accumulation in the Cells

ONOO^−^ is a very highly reactive molecule, with very short half-life. Thus, its detection in vivo is very difficult. The presence of ONOO^−^ in the tissues is often determined indirectly by measuring some reaction products (in particular nitrated proteins and lipids) using techniques such as immunodetection, chromatography and mass spectrometry [24]. Nevertheless, the same effects can be caused by other reactions, for example peroxidase activity in the presence of some RNS [30]. Thus, specific fluorescent probes for the direct detection of ONOO^−^ have been developed [31]. Figure 1 shows that the intense green fluorescence present in sycamore cells treated with FC, an index of RNS generation, is almost totally absent in cells treated with the phytotoxin in the presence of the specific ONOO^−^ scavenger urate. A very similar effect of urate on ONOO^−^ presence has been observed in cultured tobacco cells where the accumulation of ONOO^−^ was induced by heat stress [27] or elicitor treatment [29] and in *Arabidopsis thaliana* leaves where generation of ONOO^−^ occurs during the hypersensitive response induced by an avirulent *Pseudomonas syringae* strain [24]. Thus, urate appears an adequate tool to investigate the involvement of ONOO^−^ in different plant responses both in cell cultures and in whole plant.

### 3.2. Effect of FC and Urate on Cell Death and on Accumulation of TUNEL-Positive Cells

In a previous paper, we showed that FC induces in sycamore cultures the accumulation of dead cells and of cells positive to the TUNEL reaction [25]. In this paper, we show that both these effects of FC are markedly reduced by urate, especially the accumulation of TUNEL-positive cells (Figure 2 and Figure 3). At all experimental times, the percentage of TUNEL-positive cells is less than the percentage of dead cells, suggesting the presence of different forms of cell death. This is often reported in plants, where there are at least three different forms of cell death, distinguished by the organelle first involved: nucleus, mitochondrion, or vacuole [26]. While in plants a signaling role in the induction of PCD process has been clearly established for ROS and NO ([23] and references therein), the involvement of ONOO^−^ in plant cell death processes is still questioned. In fact, several authors remark that plants possess a huge network of ONOO^−^ decomposition pathways, able under physiological conditions to eliminate efficiently the reactive molecule [22]. At present, clear relationship between ONOO^−^ and plant cell death was reported in the self-incompatible pollination of olive trees where a reduction in the number of dead pollen grains and of pollen grains positive to TUNEL reaction was shown in samples treated with the specific ONOO^−^ scavenger epicatechin [32] and in tobacco cultured cells subjected to heat stress, where urate strongly reduces the accumulation of dead cells and of cells showing cytoplasmic shrinkage, another typical marker of PCD with apoptotic features [27].

### 3.3. Effect of FC and Urate on O_2_^−^ and MDA Accumulation

As previously reported, in cultured sycamore cells, FC induces oxidative stress with accumulation of ROS and MDA [33]. In this paper, we report that the FC-induced accumulation of O_2_^−^ and MDA are strongly prevented by urate (Figure 4 and Figure 5). The involvement of ONOO^−^ in the development of oxidative stress is not a surprise. In fact, due to its chemical nature, ONOO^−^ is an important biological oxidant representing a significant link between the actions of ROS and RNS [34]. Oxidative stress induced by ONOO^−^ has been reported in several plant materials, cultured cells included ([27] and references therein).

### 3.4. Effect of FC and Urate on Caspase-3-Like Activity and on the Release of Cytochrome c from Mitochondria

Apoptosis, the better-characterized form of PCD of animal cells, involves the activity of specific cysteine protease named caspases. Proteins with similar activity are present in plant cells too, which are called caspases-like or metacaspases to remark their molecular diversity to the animal counterpart [35]. FC induces a strong increase in the activity of these proteases that is almost totally prevented by urate (Figure 6). A similar effect of urate on this activity has been reported in tobacco cultured cells where the increase in caspase-like activity was induced by heat stress [27]. In this regard, it should be noted that the activity of metacaspases is regulated by nitrosylation of specific cysteine residues, a secondary protein modification depending on the presence of NO and ONOO^−^ [36]. Another typical hallmark of PCD with apoptotic features linked to caspases activity observed in both animals and plants is the release of the apoptogenic factor cytochrome *c* from mitochondria [26]. A FC-induced release of cytochrome *c* accompanied by specific DNA fragmentation (laddering) was observed previously in sycamore cells [25]. This release was shown to depend on the formation of the specific mitochondrial transition pore [28], another typical hallmark of apoptotic-like PCD. In this work, we show that the FC-dependent release of cytochrome *c* is totally prevented by urate (Figure 7). A similar effect of urate has been observed in tobacco cells subjected to heat stress [27]. In animal cells, ONOO^−^ generation is able to induce apoptosis [37]. Interestingly, ONOO^−^ can directly damage DNA, particularly by reacting with guanine to form 8-nitroguanine, and this modification can promote DNA cleavage by specific endonucleases in vivo [38].

### 3.5. Effect of FC and Urate on the Levels of Mitochondrial Hsp90, Microsomal BiP, and Cytosolic 14-3-3 Proteins

As previously reported, FC induces modification on the levels of proteins involved in the response of plants to different stresses [28]. It this work, we analyzed the effect of urate on the FC-induced modification in the level of mitochondrial Hsp90, of microsomal BiP, and of cytosolic 14-3-3s. FC only slightly reduces the level of mitochondrial Hsp90 at the first experimental time while it strongly induces Hsp90 accumulation at the second experimental time. This accumulation is largely prevented by urate (Figure 7). Other stresses, for example heat stress, are able to affect mitochondrial Hsp90 levels in plants [27,39]. Hsp90 proteins are molecular chaperones involved in the correct protein folding in every organism. Although clear indication for specific functions is still missing, several isoforms are present in different compartments (cytosol, plastids, mitochondrion, and endoplasmic reticulum) of plant cell [40]. In animals, aberrant forms of mitochondrial Hsp90 are present in cancer and neurodegenerative disorders, and their expression is consistently higher than in normal tissues where they are present at very low level [41]. Interestingly, among the protein partners of mitochondrial Hsp90, there is cyclophilin D, one of the main components of the permeability transition pore whose opening is a fundamental molecular prerequisite for the release of cytochrome *c* and the consequent induction of apoptosis [42]. In this view, it should not be forgotten that cultured plant cells could be considered in some way similar to tumor cells [43] and that FC is able to induce opening of the transition pore [28].

Urate also affects the FC-induced accumulation of BiP and 14-3-3s (Figure 7). In plants, the level of the Hsp70 molecular chaperone BiP is a general marker of endoplasmic reticulum stress. In fact, its accumulation has been reported in a number of stress conditions, heat stress included [27,44]. It should be noted that endoplasmic reticulum stress is a condition closely related to the activation of PCD programs [45]. Accumulation of regulative 14-3-3 proteins has been observed in several biotic and abiotic stress conditions [46]. As regards the involvement of ONOO^−^ on the levels of these proteins, an effect of urate in the response of *Arabidopsis thaliana* to the bacterial pathogen *Pseudomonas syringae* was reported some years ago [47]. More recently, ONOO^−^ has been shown to be implicated in the expression of genes encoding proteins involved in disease resistance of potato against *Phytophthora infestans* [48] and in the response of soybean seedling roots to cadmium [49]. Interestingly, a recent work shows that FC significantly increases the abundances of plasma membrane H^+^-ATPase and of 14-3-3s and that this increase depends on RNS treatment [50].

### 3.6. Future Research Directions

Although the specificity of urate strongly supports our hypotheses, further experiments are needed for the final confirmation of the role of ONOO^−^ in the responses induced by FC in sycamore cells. In particular, due to lack of specificity of the used dye (DAF-FM diacetate reacts with all RNS, not only with ONOO^−^) experiments performed in the presence of the NO scavenger 2-(4-carboxyphenyl)-4,4,5,5-tetramethylimidazoline-1-oxyl-3-oxide (cPTIO) would be useful to eliminate the possibility that the reported effects of FC depend on NO rather than ONOO^−^. Alternatively, it is possible to utilize more specific dye for the direct detection of ONOO^−^ such as HKGreen-2 [31].

The results reported in this manuscript also support the future utilization of FC and its derivatives as a tool in pharmacological research in animals. For example, FC appears useful to study the mechanism of action of fungal metabolite in the induction of apoptosis. In fact, many fungal metabolites show promising anticancer activities both in vitro and in model animals. However, due to lack of understanding of their mechanism of action, at present, there are no fungal-derived metabolites approved as anticancer drugs [51]. Considering its classical effect of molecular adaptor between 14-3-3s and their target proteins [52], FC can be utilized to identify proteins involved in cancer progression.

## 4. Material and Methods

### 4.1. Cell Culture Growth and Experimental Design

Sycamore *(Acer pseudoplatanus* L.) cultures were maintained as previously described [25]. Cells from cultures in the exponential phase of growth (7-day-old cultures) were utilized in the experiments. The cells were gathered by gentle centrifugation (2 min at 300 *g*) and resuspended in fresh culture medium at a final density of 106 cells mL^−1^.

**Scheme 1:**
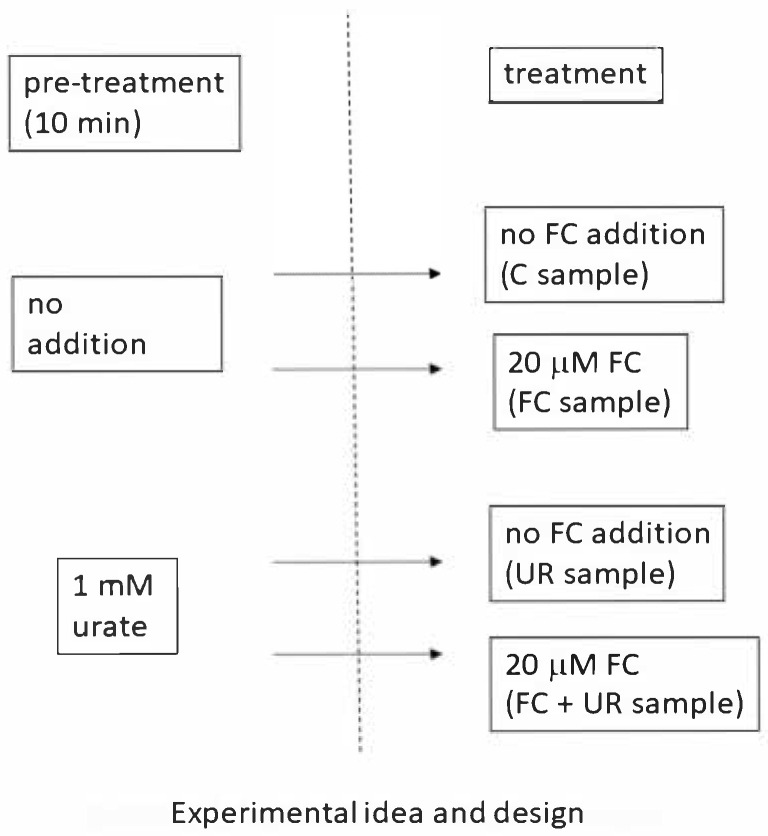
During the entire experimental period, the cells were maintained in a growth chamber at 25 °C under light on a gyratory shaker (120 rpm).

To investigate the possible involvement of ONOO^−^ in the FC-induced responses, sycamore cell suspensions were pre-treated for 10 min without or with the specific ONOO^−^ scavenger urate (1 mM final concentration, dissolved by sonication in 1 mM NaOH). After the pre-treatment, 20 µM FC was added to some of both control and urate samples to obtain the four experimental conditions: control sample, FC sample, urate sample, and FC + urate sample (see Scheme 1).

### 4.2. Cell Viability Assay and Determination of Cells Undergoing PCD

Cell death was evaluated with the vital dye Evans Blue as described using boiled cells (10 min, 100 °C) as control of 100% cell death [53]. PCD with apoptotic features was determined by counting the number of cells positive to the TUNEL reaction as described [54]. Briefly, the cells were analyzed with a fluorescein-dUTP-based cell death detection kit (Boehringer-Mannheim, Germany) following manufacturer’s instruction. At the end of the TUNEL procedure, the cells were counterstained with Hoechst 3342 (5 µg mL^−1^) in phosphate-buffered saline, pH 7.4, and analyzed with a microscope under UV-visible light. The percentage of Hoechst-labeled nuclei that were positive to TUNEL reaction was calculated from the observation of at least 1000 cells.

### 4.3. O_2_^−^ Assay and RNS Imaging

The O_2_^−^ anion generation was evaluated spectrophotometrically as reduction of sodium,3′-[1-[phenylamino-carbonyl]-3,4-tetrazolium]-bis(4-methoxy-6-nitro)benzene-sulfonic acid hydrate (XTT) to XTT formazan as described [27]. RNS accumulation in sycamore cells was visualized with the cell-permeant fluorescent probe 4-amino-5-methylamino-2′,7′-difluorofluorescein (DAF-FM) diacetate (Alexis Biochemicals, Lausen, Switzerland) as previously described [14]. Briefly, the cells, collected by gentle centrifugation (1 min at 500× *g*), were resuspended in 10 μM DAF-FM diacetate and after 15 min of incubation at room temperature they were observed with a Nikon Eclipse 90i fluorescence microscope equipped with fluorescein isothiocyanate (FITC) filter sets (Nikon, Tokyo, Japan).

### 4.4. Activity of Caspase-3-Like Proteases and Level of Lipid Peroxidation

The activity of caspase-3-like proteases was evaluated spectrophotometrically by using the caspase-3 colorimetric activity assay kit, according to the manufacturer’s instructions (BioVision Research Products, Mountain View, CA, USA) as previously described [27]. The level of lipid peroxidation was evaluated by spectrophotometrically measuring the level of malondialdehyde (MDA), a secondary end product of the oxidation of polyunsaturated fatty acids, as described [33].

### 4.5. Cell Fraction Preparation, SDS-PAGE and Protein Gel Blot Analysis

Sycamore cells were collected, homogenized, and centrifuged to obtain the different cellular fractions as described [14]. Briefly, cell homogenate was centrifuged (1000× g for 10 min) and the supernatant was centrifuged again (10,000× *g* for 15 min) to obtain a pellet representing the mitochondrial fraction. The supernatant was centrifuged (48,000× *g* for 60 min) and the obtained pellet represents the microsomal fraction. The remaining supernatant was centrifuged again (200,000× *g* for 3 h) and the resultant supernatant represented the cytosolic (soluble) fraction. SDS-PAGE and protein gel blot analyses were performed as described [14]. Immunodecoration of cytochrome *c* was performed on the cytosolic fraction with a polyclonal antibody against full-length cytochrome *c* from horse heart (Santa Cruz Biotechnology, Santa Cruz, CA, USA). Immunodecoration of cytosolic 14-3-3 proteins was performed with an antibody against the BMH1 isoform of *Saccharomyces cerevisiae*, a generous gift from Prof. Paul van Heusden, Leiden University, the Netherlands. Immunodecoration of BiP was performed on the microsomal fraction with an antibody against tobacco BiP, a generous gift from Dr. A. Vitale, Istituto di Biologia e Biotecnologia Agraria, CNR, Milano, Italy. Immunodecoration of Hsp90 was performed on the mitochondrial fraction with a polyclonal antibody against *Arabidopsis thaliana* Hsp90 (Santa Cruz Biotechnology, Santa Cruz, CA, USA). The relative abundance of immunodecorated proteins was quantified using ImageJ 1.32 J program (Wayne Rasband, National Institutes of Health, USA, http://rsb.info.nih.gov/ij/).

### 4.6. Statistical Analyses

GraphPad Prism 4 program from GraphPad Software, Inc., San Diego, CA, USA, was used for statistically analyzing the obtained results. Tukey HSD test, *p* ≤ 0.05, was used in the study.

## 5. Conclusions

Despite some recent studies, the physiological function of peroxynitrite in various cell regulatory mechanisms of plants is still controversial [21]. However, ONOO^−^ is emerging as a potential signaling molecule during the induction of defense responses against biotic and abiotic stresses. This effect could be mediated by the selective nitration of tyrosine residues in a small number of proteins and new evidence shows that nitrative modifications of nucleic acids can affect gene expression in plants, too [55].

Our results suggest a peroxynitrite involvement in some FC-induced stress responses of sycamore cells. In particular, it seems important for the FC-induced cell death form that shows apoptotic features, such as specific DNA fragmentation, caspase-3-like protease activity, and cytochrome *c* release from the mitochondrion.

## Abbreviations

BiP, Binding Protein; DAF-FM, 4-amino-5-methylamino-2′,7′-difluorofluorescein; FC, fusicoccin; NO, nitric oxide; ONOO^−^, peroxynitrite; PCD, programmed cell death; ROS, Reactive oxygen species; RNS, Reactive nitrogen species; XTT, sodium,39-[1-[phenylamino-carbonyl]-3,4-tetrazolium]-bis(4-methoxy-6-nitro) benzene-sulfonic acid hydrate.
